# Treatment of Ague

**Published:** 1890-02-01

**Authors:** 


### TREATMENT OF AGUE.

"The Oil of Eucalyptus in Some Forms of Malaria" is the title of a recent paper by Dr. J. H. Musser, assistant professor of clinical medicine in the University of Pennsylvania (*Philadelphia Medical News*, December 27). The drug was administered in some fifteen cases, and in one case only were the *bacilli malariæ* found to disappear while the oil of eucalyptus was being used. It was found that in a large number of cases of intermittent admitted into the wards of the Philadelphia Hospital that without the use of any drugs, and without any particular form of diet, but simply from change alone, or removal from the infected locality, the intermittent paroxysms grew less and less in severity and duration, and after intervals of from three to ten days would disappear entirely. In other words, under favourable circumstances the disease is self-limited. Nevertheless the writer thinks the oil of eucalyptus is especially useful in cases which may be called "habit chills." One sees persons with intermittent fever, in which, while in a measure controlled by quinine, yet even after full saturation by this drug the chills continue. Cases of this character have not been studied with regard to the presence of the bacillus, but the writer believes organisms would not be found, and that the chills recurred from habit. Oil of eucalyptus is said by the author to be serviceable in neuralgias, and in cases of general malaise depending on malaria. The dose of the oil is five drops four times daily, preferably administered in a capsule. Now there is a growing belief, not, however, without dissentients (*vide* Hospital "Retrospect," Dec. 21, "Malaria") that intermittent fevers, and so-called malarious diseases generally, are caused by the bacillus malariæ, first discovered by Kleb and Crudeli. Quinine has also been supposed to kill these bacilli, and thus to produce its good effects in ague. Admitting this is correct, and which Dr. Musser appears to believe, we do not see how oil of eucalyptus can be beneficial, for, according to the author, the bacilli do not disappear when it is used. But we have grave doubts as to quinine proving beneficial by causing the death or disappearance of the bacilli. What Dr. Musser says of ague—that the disease is self-limited—is correct, many agues disappearing spontaneously. It is on record that M. de Chambas used to cure his ague patients with water labelled *protoxide of hydrogen*. Quite recently it has been reported that Dr. Alois Ferykovy, at Nisch, finding his stock of quinine exhausted, treated his patients by rubbing the spine with simple ointment with marked success. Since that time he has usually employed this agency, and three-fourths of his patients have done well without any quinine. Many other things besides quinine have been credited with antiperiodic powers. The list includes salivation by mercury, opium, chiretta, strychnine, nitric acid, cobwebs, coffee, the hyposulphites, antipyrine, *cum multis aliis*. The explanation is probably to be found in the fact that agues often cease spontaneously. And this cessation has been attributed to the medicines employed.

